# Supplementary material for: Revised estimates of leprosy disability weights for assessing the global burden of disease: A systematic review and individual patient data meta-analysis
Source: PLoS Negl Trop Dis. 2021 Mar 2;15(3):e0009209. doi: 10.1371/journal.pntd.0009209 (PMC7954345; doi:10.1371/journal.pntd.0009209)
Supplement: S2 Table — (DOCX) [file pntd.0009209.s002.docx]

**S2 Table**

**List of similarly weighted disease sequelae**

States with similar weights to our revised grade2 leprosy estimates are highlighted in **brown**.

For grade1 is highlighted in **green**.

**GBD 2017 sequelae, health states, health state lay descriptions, and disability weights**

|  | **Sequela** | **Health state name** | **Health state lay description** | **Disability Weight** |  |
| --- | --- | --- | --- | --- | --- |
|  |  |  |  |  |  |
|  |  | Epilepsy, seizures 1- | has sudden seizures two to five times a year, with violent muscle | 0.263 |  |
|  | Idiopathic, less severe epilepsy | 11 per year | contractions and stiffness, loss of consciousness, and loss of urine or | (0.173-0.367) |  |
|  |  |  | bowel control. |  |  |
|  |  |  | is unable to hear and understand another person talking, even in a quiet |  |  |
|  | Severe hearing loss with | Hearing loss, severe, | place, and unable to take part in a phone conversation, and has | 0.261 |  |
|  | ringing due to pneumococcal |  | annoying ringing in the ears for more than 5 minutes at a time, almost |  |  |
|  | meningitis | with ringing | everyday. Difficulties with communicating and relating to others cause | (0.175-0.360) |  |
|  |  |  |  |  |  |
|  |  |  | emotional impact at times (for example worry or depression). |  |  |
|  |  |  | is unable to hear and understand another person talking, even in a quiet |  |  |
|  | Severe hearing loss with | Hearing loss, severe, | place, and unable to take part in a phone conversation, and has | 0.261 |  |
|  | ringing due to H influenzae |  | annoying ringing in the ears for more than 5 minutes at a time, almost |  |  |
|  | type B meningitis | with ringing | everyday. Difficulties with communicating and relating to others cause | (0.175-0.360) |  |
|  |  |  |  |  |  |
|  |  |  | emotional impact at times (for example worry or depression). |  |  |
|  |  |  | is unable to hear and understand another person talking, even in a quiet |  |  |
|  | Severe hearing loss with | Hearing loss, severe, | place, and unable to take part in a phone conversation, and has | 0.261 |  |
|  | ringing due to meningococcal |  | annoying ringing in the ears for more than 5 minutes at a time, almost |  |  |
|  | meningitis | with ringing | everyday. Difficulties with communicating and relating to others cause | (0.175-0.360) |  |
|  |  |  |  |  |  |
|  |  |  | emotional impact at times (for example worry or depression). |  |  |
|  |  |  | is unable to hear and understand another person talking, even in a quiet |  |  |
|  | Severe hearing loss with | Hearing loss, severe, | place, and unable to take part in a phone conversation, and has | 0.261 |  |
|  | ringing due to other bacterial |  | annoying ringing in the ears for more than 5 minutes at a time, almost |  |  |
|  | meningitis | with ringing | everyday. Difficulties with communicating and relating to others cause | (0.175-0.360) |  |
|  |  |  |  |  |  |
|  |  |  | emotional impact at times (for example worry or depression). |  |  |
|  |  |  | is unable to hear and understand another person talking, even in a quiet |  |  |
|  | Severe hearing loss with | Hearing loss, severe, | place, and unable to take part in a phone conversation, and has | 0.261 |  |
|  | ringing due to age-related and | with ringing | annoying ringing in the ears for more than 5 minutes at a time, almost | (0.175-0.360) |  |
|  | other hearing loss |  | everyday. Difficulties with communicating and relating to others cause |  |  |
|  |  |  |  |  |  |
|  |  |  | emotional impact at times (for example worry or depression). |  |  |
|  |  |  | is unable to hear and understand another person talking, even in a quiet |  |  |
|  | Severe hearing loss with | Hearing loss, severe, | place, and unable to take part in a phone conversation, and has | 0.261 |  |
|  | ringing due to other congenital |  | annoying ringing in the ears for more than 5 minutes at a time, almost |  |  |
|  | anomalies | with ringing | everyday. Difficulties with communicating and relating to others cause | (0.175-0.360) |  |
|  |  |  |  |  |  |
|  |  |  | emotional impact at times (for example worry or depression). |  |  |
|  | Severe diarrheal diseases | Diarrhea, severe | has diarrhea three or more times a day with severe belly cramps. The | 0.247 |  |
|  |  |  | person is very thirsty and feels nauseous and tired. | (0.164-0.348) |  |
|  |  |  |  |  |  |
|  |  |  |  |  |  |
|  |  |  | has frequent behavior problems, which are sometimes violent. The | 0.241 |  |
|  | Symptomatic conduct disorder | Conduct disorder | person often has difficulty interacting with other people and feels |  |  |
|  |  |  |  | (0.159-0.341) |  |
|  |  |  | irritable. |  |  |
|  |  |  |  |  |  |
|  |  | Alcohol use disorder, | drinks a lot of alcohol and sometimes has difficulty controlling the urge | 0.235 |  |
|  | Mild alcohol dependence |  | to drink. While intoxicated, the person has difficulty performing daily |  |  |
|  |  | mild | activities. | (0.160-0.327) |  |
|  | Crohn's disease with severe | Crohn disease or | has cramping abdominal pain, has diarrhea several times a day, and | 0.231 |  |
|  |  |  | feels very tired for two months every year. When the person does not |  |  |
|  | anemia | ulcerative colitis |  | (0.156-0.320) |  |
|  |  |  | have symptoms, there is anxiety about them returning. |  |  |
|  |  |  |  |  |  |
|  |  |  |  |  |  |
|  |  | Crohn disease or | has cramping abdominal pain, has diarrhea several times a day, and | 0.231 |  |
|  | Crohn's disease without anemia |  | feels very tired for two months every year. When the person does not |  |  |
|  |  | ulcerative colitis |  | (0.156-0.320) |  |
|  |  |  | have symptoms, there is anxiety about them returning. |  |  |
|  | Ulcerative colitis with mild | Crohn disease or | has cramping abdominal pain, has diarrhea several times a day, and | 0.231 |  |
|  |  |  | feels very tired for two months every year. When the person does not |  |  |
|  | anemia | ulcerative colitis |  | (0.156-0.320) |  |
|  |  |  | have symptoms, there is anxiety about them returning. |  |  |
|  |  |  |  |  |  |
|  |  |  |  |  |  |
|  | Ulcerative colitis with severe | Crohn disease or | has cramping abdominal pain, has diarrhea several times a day, and | 0.231 |  |
|  |  |  | feels very tired for two months every year. When the person does not |  |  |
|  | anemia | ulcerative colitis |  | (0.156-0.320) |  |
|  |  |  | have symptoms, there is anxiety about them returning. |  |  |
|  |  |  |  |  |  |
|  |  |  |  |  |  |
|  | Ulcerative colitis without | Crohn disease or | has cramping abdominal pain, has diarrhea several times a day, and | 0.231 |  |
|  |  |  | feels very tired for two months every year. When the person does not |  |  |
|  | anemia | ulcerative colitis |  | (0.156-0.320) |  |
|  |  |  | have symptoms, there is anxiety about them returning. |  |  |
|  |  |  |  |  |  |
|  |  |  |  |  |  |

| Crohn's disease with mild | Crohn disease or | has cramping abdominal pain, has diarrhea several times a day, and | 0.231 |
| --- | --- | --- | --- |
|  |  | feels very tired for two months every year. When the person does not |  |
| anemia | ulcerative colitis |  | (0.156-0.320) |
|  |  | have symptoms, there is anxiety about them returning. |  |
|  |  |  |  |
|  |  |  |  |
| Crohn's disease with moderate | Crohn disease or | has cramping abdominal pain, has diarrhea several times a day, and | 0.231 |
|  |  | feels very tired for two months every year. When the person does not |  |
| anemia | ulcerative colitis |  | (0.156-0.320) |
|  |  | have symptoms, there is anxiety about them returning. |  |
|  |  |  |  |
|  |  |  |  |
| Ulcerative colitis with | Crohn disease or | has cramping abdominal pain, has diarrhea several times a day, and | 0.231 |
|  |  | feels very tired for two months every year. When the person does not |  |
| moderate anemia | ulcerative colitis |  | (0.156-0.320) |
|  |  | have symptoms, there is anxiety about them returning. |  |
|  |  |  |  |
|  |  |  |  |
|  |  | has severe neck pain, and difficulty turning the head and lifting things. | 0.229 |
| Severe neck pain | Neck pain, severe | The person gets headaches and arm pain, sleeps poorly, and feels tired |  |
|  |  |  | (0.153-0.317) |
|  |  | and worried. |  |
|  |  |  |  |
| Moderate paragonimiasis due | COPD and other | has cough, wheezing and shortness of breath, even after light physical | 0.225 |
|  | chronic respiratory | activity. The person feels tired and can walk only short distances or |  |
| to food-borne trematodiases |  |  | (0.153-0.310) |
|  | problems, moderate | climb only a few stairs. |  |
|  |  |  |  |
|  |  |  |  |
| Moderate chronic obstructive | COPD and other | has cough, wheezing and shortness of breath, even after light physical | 0.225 |
|  | chronic respiratory | activity. The person feels tired and can walk only short distances or |  |
| pulmonary disease |  |  | (0.153-0.310) |
|  | problems, moderate | climb only a few stairs. |  |
|  |  |  |  |
|  |  |  |  |
|  | COPD and other | has cough, wheezing and shortness of breath, even after light physical | 0.225 |
| Moderate silicosis | chronic respiratory | activity. The person feels tired and can walk only short distances or |  |
|  |  |  | (0.153-0.310) |
|  | problems, moderate | climb only a few stairs. |  |
|  |  |  |  |
|  |  |  |  |
|  | COPD and other | has cough, wheezing and shortness of breath, even after light physical | 0.225 |
| Moderate asbestosis | chronic respiratory | activity. The person feels tired and can walk only short distances or |  |
|  |  |  | (0.153-0.310) |
|  | problems, moderate | climb only a few stairs. |  |
|  |  |  |  |
|  |  |  |  |
| Moderate coal workers | COPD and other | has cough, wheezing and shortness of breath, even after light physical | 0.225 |
|  | chronic respiratory | activity. The person feels tired and can walk only short distances or |  |
| pneumoconiosis |  |  | (0.153-0.310) |
|  | problems, moderate | climb only a few stairs. |  |
|  |  |  |  |
|  |  |  |  |
| Moderate other | COPD and other | has cough, wheezing and shortness of breath, even after light physical | 0.225 |
|  | chronic respiratory | activity. The person feels tired and can walk only short distances or |  |
| pneumoconiosis |  |  | (0.153-0.310) |
|  | problems, moderate | climb only a few stairs. |  |
|  |  |  |  |
|  |  |  |  |
| Moderate interstitial lung | COPD and other | has cough, wheezing and shortness of breath, even after light physical | 0.225 |
| disease and pulmonary | chronic respiratory | activity. The person feels tired and can walk only short distances or |  |
|  |  |  | (0.153-0.310) |
| sarcoidosis | problems, moderate | climb only a few stairs. |  |
|  |  |  |  |
|  |  |  |  |
| Moderate respiratory problems | COPD and other | has cough, wheezing and shortness of breath, even after light physical | 0.225 |
| due to motor neuron disease | chronic respiratory | activity. The person feels tired and can walk only short distances or | (0.153-0.310) |
|  | problems, moderate | climb only a few stairs. |  |
|  |  |  |  |
|  |  |  |  |
| Atrial fibrillation and flutter | Cardiac conduction |  | 0.224 |
|  | disorders and cardiac | has periods of rapid and irregular heartbeats and occasional fainting. |  |
| due to Chagas disease |  |  | (0.151-0.312) |
|  | dysrhythmias |  |  |
|  |  |  |  |
|  |  |  |  |
| Symptomatic atrial fibrillation | Cardiac conduction |  | 0.224 |
|  | disorders and cardiac | has periods of rapid and irregular heartbeats and occasional fainting. |  |
| and flutter | dysrhythmias |  | (0.151-0.312) |
|  |  |  |  |
|  |  |  |  |
| Anorexia nervosa | Anorexia nervosa | feels an overwhelming need to starve and exercises excessively to lose | 0.224 |
|  |  | weight. The person is very thin, weak and anxious. | (0.150-0.312) |
|  |  |  |  |
|  |  |  |  |
| Bulimia nervosa | Bulimia nervosa | has uncontrolled overeating followed by guilt, starving, and vomiting to | 0.223 |
|  |  | lose weight. | (0.149-0.311) |
|  |  |  |  |
| Symptomatic medication |  | has daily headaches, felt as dull pain and often lasting all day, with poor |  |
|  | Headache, | sleep, nausea and fatigue. The person takes medicine for the headaches, | 0.223 |
| overuse headache due to |  |  |  |
|  | medication overuse | which provides little relief but is needed to avoid having worse | (0.146-0.313) |
| migraine |  |  |  |
|  |  | symptoms. |  |
|  |  |  |  |
| Symptomatic medication |  | has daily headaches, felt as dull pain and often lasting all day, with poor |  |
|  | Headache, | sleep, nausea and fatigue. The person takes medicine for the headaches, | 0.223 |
| overuse headache due to |  |  |  |
|  | medication overuse | which provides little relief but is needed to avoid having worse | (0.146-0.313) |
| tension-type headache |  |  |  |
|  |  | symptoms. |  |
|  |  |  |  |

|  | Infectious disease, |  |  |
| --- | --- | --- | --- |
| Post-dengue chronic fatigue | post-acute | is always tired and easily upset. The person feels pain all over the body | 0.219 |
|  | consequences |  |  |
| syndrome |  | and is depressed. | (0.148-0.308) |
|  | (fatigue, emotional |  |  |
|  |  |  |  |
|  | lability, insomnia) |  |  |
|  |  |  |  |
|  | Infectious disease, |  |  |
| Post-Ebola chronic fatigue | post-acute | is always tired and easily upset. The person feels pain all over the body | 0.219 |
|  | consequences |  |  |
| syndrome |  | and is depressed. | (0.148-0.308) |
|  | (fatigue, emotional |  |  |
|  |  |  |  |
|  | lability, insomnia) |  |  |
|  |  |  |  |
|  |  | cannot hear at all in any situation, including even the loudest sounds, |  |
| Complete hearing loss due to | Hearing loss, | and cannot communicate verbally or use a phone. Difficulties with | 0.215 |
| pneumococcal meningitis | complete | communicating and relating to others often cause worry, depression or | (0.144-0.307) |
|  |  | loneliness. |  |
|  |  | cannot hear at all in any situation, including even the loudest sounds, |  |
| Complete hearing loss due to H | Hearing loss, | and cannot communicate verbally or use a phone. Difficulties with | 0.215 |
| influenzae type B meningitis | complete | communicating and relating to others often cause worry, depression or | (0.144-0.307) |
|  |  | loneliness. |  |
|  |  | cannot hear at all in any situation, including even the loudest sounds, |  |
| Complete hearing loss due to | Hearing loss, | and cannot communicate verbally or use a phone. Difficulties with | 0.215 |
| meningococcal meningitis | complete | communicating and relating to others often cause worry, depression or | (0.144-0.307) |
|  |  | loneliness. |  |
|  |  | cannot hear at all in any situation, including even the loudest sounds, |  |
| Complete hearing loss due to | Hearing loss, | and cannot communicate verbally or use a phone. Difficulties with | 0.215 |
| other bacterial meningitis | complete | communicating and relating to others often cause worry, depression or | (0.144-0.307) |
|  |  | loneliness. |  |
| Complete hearing loss due to |  | cannot hear at all in any situation, including even the loudest sounds, |  |
|  | Hearing loss, | and cannot communicate verbally or use a phone. Difficulties with | 0.215 |
| age-related and other hearing |  |  |  |
|  | complete | communicating and relating to others often cause worry, depression or | (0.144-0.307) |
| loss |  |  |  |
|  |  | loneliness. |  |
|  |  |  |  |
|  |  | cannot hear at all in any situation, including even the loudest sounds, |  |
| Complete hearing loss due to | Hearing loss, | and cannot communicate verbally or use a phone. Difficulties with | 0.215 |
| other congenital anomalies | complete | communicating and relating to others often cause worry, depression or | (0.144-0.307) |
|  |  | loneliness. |  |
|  |  | is unable to hear and understand another person talking, even in a quiet |  |
| Profound hearing loss due to | Hearing loss, | place, is unable to take part in a phone conversation, and has great | 0.204 |
|  |  | difficulty hearing anything in any other situation. Difficulties with |  |
| pneumococcal meningitis | profound |  | (0.134-0.288) |
|  |  | communicating and relating to other soften cause worry, depression, |  |
|  |  |  |  |
|  |  | and loneliness. |  |
|  |  | is unable to hear and understand another person talking, even in a quiet |  |
| Profound hearing loss due to H | Hearing loss, | place, is unable to take part in a phone conversation, and has great | 0.204 |
|  |  | difficulty hearing anything in any other situation. Difficulties with |  |
| influenzae type B meningitis | profound |  | (0.134-0.288) |
|  |  | communicating and relating to other soften cause worry, depression, |  |
|  |  |  |  |
|  |  | and loneliness. |  |
|  |  | is unable to hear and understand another person talking, even in a quiet |  |
| Profound hearing loss due to | Hearing loss, | place, is unable to take part in a phone conversation, and has great | 0.204 |
|  |  | difficulty hearing anything in any other situation. Difficulties with |  |
| meningococcal meningitis | profound |  | (0.134-0.288) |
|  |  | communicating and relating to other soften cause worry, depression, |  |
|  |  |  |  |
|  |  | and loneliness. |  |
|  |  | is unable to hear and understand another person talking, even in a quiet |  |
| Profound hearing loss due to | Hearing loss, | place, is unable to take part in a phone conversation, and has great | 0.204 |
|  |  | difficulty hearing anything in any other situation. Difficulties with |  |
| other bacterial meningitis | profound |  | (0.134-0.288) |
|  |  | communicating and relating to other soften cause worry, depression, |  |
|  |  |  |  |
|  |  | and loneliness. |  |
|  |  | is unable to hear and understand another person talking, even in a quiet |  |
| Profound hearing loss due to | Hearing loss, | place, is unable to take part in a phone conversation, and has great | 0.204 |
| age-related and other hearing |  | difficulty hearing anything in any other situation. Difficulties with |  |
|  | profound |  | (0.134-0.288) |
| loss |  | communicating and relating to other soften cause worry, depression, |  |
|  |  |  |  |
|  |  | and loneliness. |  |
|  |  | is unable to hear and understand another person talking, even in a quiet |  |
| Profound hearing loss due to | Hearing loss, | place, is unable to take part in a phone conversation, and has great | 0.204 |
|  |  | difficulty hearing anything in any other situation. Difficulties with |  |
| other congenital anomalies | profound |  | (0.134-0.288) |
|  |  | communicating and relating to other soften cause worry, depression, |  |
|  |  |  |  |
|  |  | and loneliness. |  |
| Neurological problems due to | Motor plus cognitive | has some difficulty in moving around, holding objects, dressing and | 0.203 |
|  | impairments, | sitting upright, but can walk without help. The person has low |  |
| adult tertiary syphilis |  |  | (0.134-0.290) |
|  | moderate | intelligence and is slow in learning to speak and to do simple tasks. |  |
|  |  |  |  |
|  |  |  |  |
| Moderate motor plus cognitive | Motor plus cognitive | has some difficulty in moving around, holding objects, dressing and | 0.203 |
| impairments due to | impairments, | sitting upright, but can walk without help. The person has low |  |
|  |  |  | (0.134-0.290) |
| pneumococcal meningitis | moderate | intelligence and is slow in learning to speak and to do simple tasks. |  |
|  |  |  |  |
|  |  |  |  |
| Moderate motor plus cognitive | Motor plus cognitive | has some difficulty in moving around, holding objects, dressing and | 0.203 |
| impairments due to H | impairments, | sitting upright, but can walk without help. The person has low |  |
|  |  |  | (0.134-0.290) |
| influenzae type B meningitis | moderate | intelligence and is slow in learning to speak and to do simple tasks. |  |
|  |  |  |  |
|  |  |  |  |
| Moderate motor plus cognitive | Motor plus cognitive | has some difficulty in moving around, holding objects, dressing and | 0.203 |
| impairments due to | impairments, | sitting upright, but can walk without help. The person has low |  |
|  |  |  | (0.134-0.290) |
| meningococcal meningitis | moderate | intelligence and is slow in learning to speak and to do simple tasks. |  |
|  |  |  |  |
|  |  |  |  |

| Moderate motor plus cognitive | Motor plus cognitive | has some difficulty in moving around, holding objects, dressing and | 0.203 |
| --- | --- | --- | --- |
| impairments due to other | impairments, | sitting upright, but can walk without help. The person has low |  |
|  |  |  | (0.134-0.290) |
| bacterial meningitis | moderate | intelligence and is slow in learning to speak and to do simple tasks. |  |
|  |  |  |  |
|  |  |  |  |
| Moderate motor plus cognitive | Motor plus cognitive | has some difficulty in moving around, holding objects, dressing and | 0.203 |
| impairments due to | impairments, | sitting upright, but can walk without help. The person has low |  |
|  |  |  | (0.134-0.290) |
| encephalitis | moderate | intelligence and is slow in learning to speak and to do simple tasks. |  |
|  |  |  |  |
|  |  |  |  |
| Moderate motor impairment | Motor plus cognitive | has some difficulty in moving around, holding objects, dressing and | 0.203 |
| and moderate intellectual | impairments, | sitting upright, but can walk without help. The person has low |  |
|  |  |  | (0.134-0.290) |
| disability due to encephalocele | moderate | intelligence and is slow in learning to speak and to do simple tasks. |  |
|  |  |  |  |
|  |  |  |  |
| Moderate motor impairment | Motor plus cognitive | has some difficulty in moving around, holding objects, dressing and | 0.203 |
| and moderate intellectual | impairments, | sitting upright, but can walk without help. The person has low |  |
|  |  |  | (0.134-0.290) |
| disability due to spina bifida | moderate | intelligence and is slow in learning to speak and to do simple tasks. |  |
|  |  |  |  |
|  |  |  |  |
| Profound idiopathic | Intellectual disability | has very low intelligence, has almost no language, and does not | 0.200 |
| developmental intellectual | / mental retardation, | understand even the most basic requests or instructions. The person |  |
|  |  |  | (0.133-0.283) |
| disability | profound | requires constant supervision and help for all activities. |  |
|  |  |  |  |
| Profound intellectual disability | Intellectual disability | has very low intelligence, has almost no language, and does not | 0.200 |
|  | / mental retardation, | understand even the most basic requests or instructions. The person |  |
| due to encephalocele | profound | requires constant supervision and help for all activities. | (0.133-0.283) |
|  |  |  |  |
| Profound intellectual disability | Intellectual disability | has very low intelligence, has almost no language, and does not | 0.200 |
|  | / mental retardation, | understand even the most basic requests or instructions. The person |  |
| due to Down syndrome | profound | requires constant supervision and help for all activities. | (0.133-0.283) |
|  |  |  |  |
| Profound intellectual disability | Intellectual disability | has very low intelligence, has almost no language, and does not | 0.200 |
| due to other chromosomal | / mental retardation, | understand even the most basic requests or instructions. The person |  |
|  |  |  | (0.133-0.283) |
| abnormalities | profound | requires constant supervision and help for all activities. |  |
|  |  |  |  |
| Moderate skin disease due to | Disfigurement, level | has a visible physical deformity that is sore and itchy. Other people | 0.188 |
|  |  | stare and comment, which causes the person to worry. The person has |  |
| onchocerciasis | 2, with itch/pain | trouble sleeping and concentrating. | (0.125-0.267) |
| Severe skin disease due to | Disfigurement, level | has a visible physical deformity that is sore and itchy. Other people | 0.188 |
| onchocerciasis | 2, with itch/pain | stare and comment, which causes the person to worry. The person has | (0.125-0.267) |
|  |  | trouble sleeping and concentrating. |  |
|  |  |  |  |
| Moderate pain due to Guinea | Disfigurement, level | has a visible physical deformity that is sore and itchy. Other people | 0.188 |
|  |  | stare and comment, which causes the person to worry. The person has |  |
| worm emergence | 2, with itch/pain | trouble sleeping and concentrating. | (0.125-0.267) |
|  | Disfigurement, level | has a visible physical deformity that is sore and itchy. Other people | 0.188 |
| Moderate atopic dermatitis |  | stare and comment, which causes the person to worry. The person has |  |
|  | 2, with itch/pain | trouble sleeping and concentrating. | (0.125-0.267) |
| Moderate contact dermatitis | Disfigurement, level | has a visible physical deformity that is sore and itchy. Other people | 0.188 |
|  | 2, with itch/pain | stare and comment, which causes the person to worry. The person has | (0.125-0.267) |
|  |  | trouble sleeping and concentrating. |  |
|  |  |  |  |
|  | Disfigurement, level | has a visible physical deformity that is sore and itchy. Other people | 0.188 |
| Moderate psoriasis |  | stare and comment, which causes the person to worry. The person has |  |
|  | 2, with itch/pain | trouble sleeping and concentrating. | (0.125-0.267) |
|  | Disfigurement, level | has a visible physical deformity that is sore and itchy. Other people | 0.188 |
| Severe urticaria |  | stare and comment, which causes the person to worry. The person has |  |
|  | 2, with itch/pain | trouble sleeping and concentrating. | (0.125-0.267) |
| Moderate decubitus ulcer | Disfigurement, level | has a visible physical deformity that is sore and itchy. Other people | 0.188 |
|  | 2, with itch/pain | stare and comment, which causes the person to worry. The person has | (0.125-0.267) |
|  |  | trouble sleeping and concentrating. |  |
|  |  |  |  |
| Disfigurement level 2 with pain | Disfigurement, level | has a visible physical deformity that is sore and itchy. Other people | 0.188 |
| due to other congenital |  | stare and comment, which causes the person to worry. The person has |  |
| musculoskeletal anomalies | 2, with itch/pain | trouble sleeping and concentrating. | (0.125-0.267) |
|  |  |  |  |
|  |  |  |  |
| Disfigurement level 2 with pain | Disfigurement, level | has a visible physical deformity that is sore and itchy. Other people | 0.188 |
| due to congenital limb |  | stare and comment, which causes the person to worry. The person has |  |
| deficiency | 2, with itch/pain | trouble sleeping and concentrating. | (0.125-0.267) |
| Moderate diarrheal diseases | Diarrhea, moderate | has diarrhea three or more times a day, with painful cramps in the belly | 0.188 |
|  |  | and feeling thirsty | (0.125-0.264) |
|  |  |  |  |
| Blindness due to | Distance vision | is completely blind, which causes great difficulty in some daily | 0.187 |
|  |  | activities, worry and anxiety, and great difficulty going outside the |  |
| onchocerciasis | blindness |  | (0.124-0.260) |
|  |  | home without assistance. |  |
|  |  |  |  |
|  | Distance vision | is completely blind, which causes great difficulty in some daily | 0.187 |
| Blindness due to trachoma |  | activities, worry and anxiety, and great difficulty going outside the |  |
|  | blindness |  | (0.124-0.260) |
|  |  | home without assistance. |  |
|  |  |  |  |
| Blindness due to pneumococcal | Distance vision | is completely blind, which causes great difficulty in some daily | 0.187 |
|  |  | activities, worry and anxiety, and great difficulty going outside the |  |
| meningitis | blindness |  | (0.124-0.260) |
|  |  | home without assistance. |  |
|  |  |  |  |
| Blindness due to H influenzae | Distance vision | is completely blind, which causes great difficulty in some daily | 0.187 |
|  |  | activities, worry and anxiety, and great difficulty going outside the |  |
| type B meningitis | blindness |  | (0.124-0.260) |
|  |  | home without assistance. |  |
|  |  |  |  |
| Blindness due to | Distance vision | is completely blind, which causes great difficulty in some daily | 0.187 |
|  |  | activities, worry and anxiety, and great difficulty going outside the |  |
| meningococcal meningitis | blindness |  | (0.124-0.260) |
|  |  | home without assistance. |  |
|  |  |  |  |
| Blindness due to other bacterial | Distance vision | is completely blind, which causes great difficulty in some daily | 0.187 |
|  |  | activities, worry and anxiety, and great difficulty going outside the |  |
| meningitis | blindness |  | (0.124-0.260) |
|  |  | home without assistance. |  |
|  |  |  |  |

|  | Distance vision | is completely blind, which causes great difficulty in some daily | 0.187 |
| --- | --- | --- | --- |
| Blindness due to encephalitis |  | activities, worry and anxiety, and great difficulty going outside the |  |
|  | blindness |  | (0.124-0.260) |
|  |  | home without assistance. |  |
|  |  |  |  |
| Blindness due to retinopathy of | Distance vision | is completely blind, which causes great difficulty in some daily | 0.187 |
|  |  | activities, worry and anxiety, and great difficulty going outside the |  |
| prematurity | blindness |  | (0.124-0.260) |
|  |  | home without assistance. |  |
|  |  |  |  |
| Blindness due to vitamin A | Distance vision | is completely blind, which causes great difficulty in some daily | 0.187 |
|  |  | activities, worry and anxiety, and great difficulty going outside the |  |
| deficiency | blindness |  | (0.124-0.260) |
|  |  | home without assistance. |  |
|  |  |  |  |
| Blindness due to diabetes | Distance vision | is completely blind, which causes great difficulty in some daily | 0.187 |
|  |  | activities, worry and anxiety, and great difficulty going outside the |  |
| mellitus type 1 | blindness |  | (0.124-0.260) |
|  |  | home without assistance. |  |
|  |  |  |  |
| Blindness due to diabetes | Distance vision | is completely blind, which causes great difficulty in some daily | 0.187 |
|  |  | activities, worry and anxiety, and great difficulty going outside the |  |
| mellitus type 2 | blindness |  | (0.124-0.260) |
|  |  | home without assistance. |  |
|  |  |  |  |
|  | Distance vision | is completely blind, which causes great difficulty in some daily | 0.187 |
| Blindness due to glaucoma |  | activities, worry and anxiety, and great difficulty going outside the |  |
|  | blindness |  | (0.124-0.260) |
|  |  | home without assistance. |  |
|  |  |  |  |
|  | Distance vision | is completely blind, which causes great difficulty in some daily | 0.187 |
| Blindness due to cataract |  | activities, worry and anxiety, and great difficulty going outside the |  |
|  | blindness |  | (0.124-0.260) |
|  |  | home without assistance. |  |
|  |  |  |  |
| Blindness due to macular | Distance vision | is completely blind, which causes great difficulty in some daily | 0.187 |
|  |  | activities, worry and anxiety, and great difficulty going outside the |  |
| degeneration | blindness |  | (0.124-0.260) |
|  |  | home without assistance. |  |
|  |  |  |  |
| Blindness due to uncorrected | Distance vision | is completely blind, which causes great difficulty in some daily | 0.187 |
|  |  | activities, worry and anxiety, and great difficulty going outside the |  |
| refractive error | blindness |  | (0.124-0.260) |
|  |  | home without assistance. |  |
|  |  |  |  |
| Blindness due to other vision | Distance vision | is completely blind, which causes great difficulty in some daily | 0.187 |
|  |  | activities, worry and anxiety, and great difficulty going outside the |  |
| loss | blindness |  | (0.124-0.260) |
|  |  | home without assistance. |  |
|  |  |  |  |
| Severe vision impairment due | Distance vision, | has severe vision loss, which causes difficulty in daily activities, some | 0.184 |
|  |  | emotional impact (for example worry), and some difficulty going |  |
| to onchocerciasis | severe impairment |  | (0.125-0.258) |
|  |  | outside the home without assistance. |  |
|  |  |  |  |
| Severe vision impairment due | Distance vision, | has severe vision loss, which causes difficulty in daily activities, some | 0.184 |
|  |  | emotional impact (for example worry), and some difficulty going |  |
| to trachoma | severe impairment |  | (0.125-0.258) |
|  |  | outside the home without assistance. |  |
|  |  |  |  |
| Severe vision impairment due | Distance vision, | has severe vision loss, which causes difficulty in daily activities, some | 0.184 |
|  |  | emotional impact (for example worry), and some difficulty going |  |
| to pneumococcal meningitis | severe impairment |  | (0.125-0.258) |
|  |  | outside the home without assistance. |  |
|  |  |  |  |
| Severe vision impairment due | Distance vision, | has severe vision loss, which causes difficulty in daily activities, some | 0.184 |
| to H influenza type B |  | emotional impact (for example worry), and some difficulty going |  |
|  | severe impairment |  | (0.125-0.258) |
| meningitis |  | outside the home without assistance. |  |
|  |  |  |  |
| Severe vision impairment due | Distance vision, | has severe vision loss, which causes difficulty in daily activities, some | 0.184 |
|  |  | emotional impact (for example worry), and some difficulty going |  |
| to meningococcal meningitis | severe impairment |  | (0.125-0.258) |
|  |  | outside the home without assistance. |  |
|  |  |  |  |
| Severe vision impairment due | Distance vision, | has severe vision loss, which causes difficulty in daily activities, some | 0.184 |
|  |  | emotional impact (for example worry), and some difficulty going |  |
| to other bacterial meningitis | severe impairment |  | (0.125-0.258) |
|  |  | outside the home without assistance. |  |
|  |  |  |  |
| Severe vision impairment due | Distance vision, | has severe vision loss, which causes difficulty in daily activities, some | 0.184 |
|  |  | emotional impact (for example worry), and some difficulty going |  |
| to encephalitis | severe impairment |  | (0.125-0.258) |
|  |  | outside the home without assistance. |  |
|  |  |  |  |
| Severe vision impairment due | Distance vision, | has severe vision loss, which causes difficulty in daily activities, some | 0.184 |
|  |  | emotional impact (for example worry), and some difficulty going |  |
| to retinopathy of prematurity | severe impairment |  | (0.125-0.258) |
|  |  | outside the home without assistance. |  |
|  |  |  |  |
| Severe vision impairment loss | Distance vision, | has severe vision loss, which causes difficulty in daily activities, some | 0.184 |
|  |  | emotional impact (for example worry), and some difficulty going |  |
| due to vitamin A deficiency | severe impairment |  | (0.125-0.258) |
|  |  | outside the home without assistance. |  |
|  |  |  |  |
| Severe vision impairment due | Distance vision, | has severe vision loss, which causes difficulty in daily activities, some | 0.184 |
|  |  | emotional impact (for example worry), and some difficulty going |  |
| to diabetes mellitus type 1 | severe impairment |  | (0.125-0.258) |
|  |  | outside the home without assistance. |  |
|  |  |  |  |
| Severe vision impairment due | Distance vision, | has severe vision loss, which causes difficulty in daily activities, some | 0.184 |
|  |  | emotional impact (for example worry), and some difficulty going |  |
| to diabetes mellitus type 2 | severe impairment |  | (0.125-0.258) |
|  |  | outside the home without assistance. |  |
|  |  |  |  |
| Severe vision impairment due | Distance vision, | has severe vision loss, which causes difficulty in daily activities, some | 0.184 |
|  |  | emotional impact (for example worry), and some difficulty going |  |
| to glaucoma | severe impairment |  | (0.125-0.258) |
|  |  | outside the home without assistance. |  |
|  |  |  |  |
| Severe vision impairment due | Distance vision, | has severe vision loss, which causes difficulty in daily activities, some | 0.184 |
|  |  | emotional impact (for example worry), and some difficulty going |  |
| to cataract | severe impairment |  | (0.125-0.258) |
|  |  | outside the home without assistance. |  |
|  |  |  |  |
| Severe vision impairment due | Distance vision, | has severe vision loss, which causes difficulty in daily activities, some | 0.184 |
|  |  | emotional impact (for example worry), and some difficulty going |  |
| to other vision loss | severe impairment |  | (0.125-0.258) |
|  |  | outside the home without assistance. |  |
|  |  |  |  |
| Mild multiple sclerosis | Multiple sclerosis, | has mild loss of feeling in one hand, is a little unsteady while walking, | 0.183 |
|  | mild | has slight loss of vision in one eye, and often needs to urinate urgently. | (0.124-0.253) |
|  |  |  |  |
|  |  |  |  |
| Severe heart failure due to | Heart failure, severe | is short of breath and feels tired when at rest. The person avoids any | 0.179 |
| Chagas disease |  | physical activity, for fear of worsening the breathing problems. | (0.122-0.251) |
|  |  |  |  |

| Severe heart failure due to | Heart failure, severe | is short of breath and feels tired when at rest. The person avoids any | 0.179 |
| --- | --- | --- | --- |
| rheumatic heart disease |  | physical activity, for fear of worsening the breathing problems. | (0.122-0.251) |
|  |  |  |  |
| Severe heart failure due to | Heart failure, severe | is short of breath and feels tired when at rest. The person avoids any | 0.179 |
| ischemic heart disease |  | physical activity, for fear of worsening the breathing problems. | (0.122-0.251) |
|  |  |  |  |
| Severe heart failure due to | Heart failure, severe | is short of breath and feels tired when at rest. The person avoids any | 0.179 |
| hypertensive heart disease |  | physical activity, for fear of worsening the breathing problems. | (0.122-0.251) |
|  |  |  |  |
| Severe heart failure due to | Heart failure, severe | is short of breath and feels tired when at rest. The person avoids any | 0.179 |
| calcific aortic valve disease |  | physical activity, for fear of worsening the breathing problems. | (0.122-0.251) |
|  |  |  |  |
| Severe heart failure due to |  | is short of breath and feels tired when at rest. The person avoids any | 0.179 |
| degenerative mitral valve | Heart failure, severe |  |  |
|  |  | physical activity, for fear of worsening the breathing problems. | (0.122-0.251) |
| disease |  |  |  |
| Severe heart failure due to |  | is short of breath and feels tired when at rest. The person avoids any | 0.179 |
| other non-rheumatic valve | Heart failure, severe |  |  |
|  |  | physical activity, for fear of worsening the breathing problems. | (0.122-0.251) |
| disease |  |  |  |
| Severe heart failure due to | Heart failure, severe | is short of breath and feels tired when at rest. The person avoids any | 0.179 |
| myocarditis |  | physical activity, for fear of worsening the breathing problems. | (0.122-0.251) |
|  |  |  |  |
| Severe heart failure due to | Heart failure, severe | is short of breath and feels tired when at rest. The person avoids any | 0.179 |
| alcoholic cardiomyopathy |  | physical activity, for fear of worsening the breathing problems. | (0.122-0.251) |
|  |  |  |  |
| Severe heart failure due to | Heart failure, severe | is short of breath and feels tired when at rest. The person avoids any | 0.179 |
| other cardiomyopathy |  | physical activity, for fear of worsening the breathing problems. | (0.122-0.251) |
| Severe heart failure due to | Heart failure, severe | is short of breath and feels tired when at rest. The person avoids any | 0.179 |
| endocarditis |  | physical activity, for fear of worsening the breathing problems. | (0.122-0.251) |
| Severe other cardiovascular | Heart failure, severe | is short of breath and feels tired when at rest. The person avoids any | 0.179 |
| diseases |  | physical activity, for fear of worsening the breathing problems. | (0.122-0.251) |
|  |  |  |  |
| Severe heart failure due to | Heart failure, severe | is short of breath and feels tired when at rest. The person avoids any | 0.179 |
| other cardiovascular diseases |  | physical activity, for fear of worsening the breathing problems. | (0.122-0.251) |
|  |  |  |  |
| Severe heart failure due to | Heart failure, severe | is short of breath and feels tired when at rest. The person avoids any | 0.179 |
| thalassemias |  | physical activity, for fear of worsening the breathing problems. | (0.122-0.251) |
|  |  |  |  |
| Severe heart failure due to | Heart failure, severe | is short of breath and feels tired when at rest. The person avoids any | 0.179 |
| G6PD deficiency |  | physical activity, for fear of worsening the breathing problems. | (0.122-0.251) |
|  |  |  |  |
| Severe heart failure due to |  | is short of breath and feels tired when at rest. The person avoids any | 0.179 |
| other hemoglobinopathies and | Heart failure, severe |  |  |
|  |  | physical activity, for fear of worsening the breathing problems. | (0.122-0.251) |
| hemolytic anemias |  |  |  |
| Severe heart failure due to |  | is short of breath and feels tired when at rest. The person avoids any | 0.179 |
| endocrine, metabolic, blood, | Heart failure, severe |  |  |
| and immune disorders |  | physical activity, for fear of worsening the breathing problems. | (0.122-0.251) |
|  |  |  |  |
|  |  |  |  |
| Severe fetal alcohol syndrome | Fetal alcohol | is very slow in developing physically and mentally, which causes great | 0.179 |
|  | syndrome, severe | difficulty in daily activities. | (0.119-0.257) |
| Cirrhosis and other chronic | Decompensated | has a swollen belly and swollen legs. The person feels weakness, | 0.178 |
| liver diseases due to hepatitis |  |  |  |
|  | cirrhosis of the liver | fatigue and loss of appetite. | (0.123-0.250) |
| B, decompensated |  |  |  |
|  |  |  |  |
|  |  |  |  |
| Cirrhosis and other chronic | Decompensated | has a swollen belly and swollen legs. The person feels weakness, | 0.178 |
| liver diseases due to hepatitis |  |  |  |
|  | cirrhosis of the liver | fatigue and loss of appetite. | (0.123-0.250) |
| C, decompensated |  |  |  |
|  |  |  |  |
|  |  |  |  |
| Cirrhosis and other chronic | Decompensated | has a swollen belly and swollen legs. The person feels weakness, | 0.178 |
| liver diseases due to alcohol, |  |  |  |
|  | cirrhosis of the liver | fatigue and loss of appetite. | (0.123-0.250) |
| decompensated |  |  |  |
|  |  |  |  |
|  |  |  |  |
| Cirrhosis and other chronic | Decompensated | has a swollen belly and swollen legs. The person feels weakness, | 0.178 |
| liver diseases due to NASH, |  |  |  |
|  | cirrhosis of the liver | fatigue and loss of appetite. | (0.123-0.250) |
| decompensated |  |  |  |
|  |  |  |  |
|  |  |  |  |
| Cirrhosis and other chronic | Decompensated | has a swollen belly and swollen legs. The person feels weakness, | 0.178 |
| liver diseases due to other |  |  |  |
|  | cirrhosis of the liver | fatigue and loss of appetite. | (0.123-0.250) |
| cause, decompensated |  |  |  |
|  |  |  |  |
|  |  |  |  |
| Severe angina due to ischemic | Angina pectoris, | has chest pain that occurs with minimal physical activity, such as | 0.167 |
|  |  | walking only a short distance. After a brief rest, the pain goes away. |  |
| heart disease | severe |  | (0.110-0.240) |
|  |  | The person avoids most physical activities because of the pain. |  |
|  |  |  |  |
|  |  |  |  |
|  | Musculoskeletal | has severe pain in the leg, which makes the person limp and causes a lot | 0.165 |
| Severe osteoarthritis of the hip | problems, lower | of difficulty walking, standing, lifting and carrying heavy things, getting |  |
|  |  |  | (0.112-0.232) |
|  | limbs, severe | up and down, and sleeping. |  |
|  |  |  |  |
| Severe osteoarthritis of the | Musculoskeletal | has severe pain in the leg, which makes the person limp and causes a lot | 0.165 |
|  | problems, lower | of difficulty walking, standing, lifting and carrying heavy things, getting |  |
| knee |  |  | (0.112-0.232) |
|  | limbs, severe | up and down, and sleeping. |  |
|  |  |  |  |

| Other musculoskeletal | Musculoskeletal | has severe pain in the leg, which makes the person limp and causes a lot | 0.165 |
| --- | --- | --- | --- |
|  | problems, lower | of difficulty walking, standing, lifting and carrying heavy things, getting |  |
| disorders severity level 4 |  |  | (0.112-0.232) |
|  | limbs, severe | up and down, and sleeping. |  |
|  |  |  |  |
| Severe idiopathic | Intellectual disability | has very low intelligence and cannot speak more than a few words, | 0.160 |
| developmental intellectual | / mental retardation, | needs constant supervision and help with most daily activities, and can |  |
|  |  |  | (0.107-0.226) |
| disability | severe | do only the simplest tasks. |  |
|  |  |  |  |
|  |  |  |  |
| Severe intellectual disability | Intellectual disability | has very low intelligence and cannot speak more than a few words, | 0.160 |
|  | / mental retardation, | needs constant supervision and help with most daily activities, and can |  |
| due to encephalocele |  |  | (0.107-0.226) |
|  | severe | do only the simplest tasks. |  |
|  |  |  |  |
|  |  |  |  |
| Severe intellectual disability | Intellectual disability | has very low intelligence and cannot speak more than a few words, | 0.160 |
|  | / mental retardation, | needs constant supervision and help with most daily activities, and can |  |
| due to Down syndrome |  |  | (0.107-0.226) |
|  | severe | do only the simplest tasks. |  |
|  |  |  |  |
|  |  |  |  |
| Severe intellectual disability | Intellectual disability | has very low intelligence and cannot speak more than a few words, | 0.160 |
| due to other chromosomal | / mental retardation, | needs constant supervision and help with most daily activities, and can |  |
|  |  |  | (0.107-0.226) |
| abnormalities | severe | do only the simplest tasks. |  |
|  |  |  |  |
|  |  |  |  |
| Severe endocrine, metabolic, | Thrombocytopenic | easily bruises and sometimes bleeds from the gums and nose; feels | 0.159 |
| blood, and immune disorders | purpura | weak and has some difficulty with daily activities. | (0.106-0.226) |
|  |  |  |  |
|  |  | is unable to hear and understand another person talking, even in a quiet |  |
| Severe hearing loss due to | Hearing loss, severe | place, and unable to take part in a phone conversation. Difficulties with | 0.158 |
| pneumococcal meningitis |  | communicating and relating to others cause emotional impact at times | (0.105-0.227) |
|  |  |  |  |
|  |  | (for example worry or depression). |  |
|  |  |  |  |
|  |  | is unable to hear and understand another person talking, even in a quiet |  |
| Severe hearing loss due to H | Hearing loss, severe | place, and unable to take part in a phone conversation. Difficulties with | 0.158 |
| influenzae type B meningitis |  | communicating and relating to others cause emotional impact at times | (0.105-0.227) |
|  |  |  |  |
|  |  | (for example worry or depression). |  |
|  |  |  |  |
|  |  | is unable to hear and understand another person talking, even in a quiet |  |
| Severe hearing loss due to | Hearing loss, severe | place, and unable to take part in a phone conversation. Difficulties with | 0.158 |
| meningococcal meningitis |  | communicating and relating to others cause emotional impact at times | (0.105-0.227) |
|  |  |  |  |
|  |  | (for example worry or depression). |  |
|  |  |  |  |
|  |  | is unable to hear and understand another person talking, even in a quiet |  |
| Severe hearing loss due to | Hearing loss, severe | place, and unable to take part in a phone conversation. Difficulties with | 0.158 |
| other bacterial meningitis |  | communicating and relating to others cause emotional impact at times | (0.105-0.227) |
|  |  |  |  |
|  |  | (for example worry or depression). |  |
|  |  |  |  |
|  |  | is unable to hear and understand another person talking, even in a quiet |  |
| Severe hearing loss due to age- | Hearing loss, severe | place, and unable to take part in a phone conversation. Difficulties with | 0.158 |
| related and other hearing loss |  | communicating and relating to others cause emotional impact at times | (0.105-0.227) |
|  |  | (for example worry or depression). |  |
|  |  |  |  |
|  |  | is unable to hear and understand another person talking, even in a quiet |  |
| Severe hearing loss due to | Hearing loss, severe | place, and unable to take part in a phone conversation. Difficulties with | 0.158 |
| other congenital anomalies |  | communicating and relating to others cause emotional impact at times | (0.105-0.227) |
|  |  |  |  |
|  |  | (for example worry or depression). |  |
|  |  |  |  |
| Severe anemia due to malaria | Anemia, severe | feels very weak, tired and short of breath, and has problems with | 0.149 |
| vivax (PvPR) |  | activities that require physical effort or deep concentration. | (0.101-0.209) |
|  |  |  |  |
|  |  |  |  |
| Severe anemia due to malaria | Anemia, severe | feels very weak, tired and short of breath, and has problems with | 0.149 |
| parasitemia (PfPR) |  | activities that require physical effort or deep concentration. | (0.101-0.209) |
|  |  |  |  |
|  |  |  |  |
| Severe anemia due to | Anemia, severe | feels very weak, tired and short of breath, and has problems with | 0.149 |
| schistosomiasis |  | activities that require physical effort or deep concentration. | (0.101-0.209) |
|  |  |  |  |
|  |  |  |  |
| Severe anemia due to | Anemia, severe | feels very weak, tired and short of breath, and has problems with | 0.149 |
| hookworm disease |  | activities that require physical effort or deep concentration. | (0.101-0.209) |
|  |  |  |  |
|  |  |  |  |
| Severe anemia due to other | Anemia, severe | feels very weak, tired and short of breath, and has problems with | 0.149 |
| neglected tropical diseases |  | activities that require physical effort or deep concentration. | (0.101-0.209) |
|  |  |  |  |
|  |  |  |  |
| Severe anemia due to other | Anemia, severe | feels very weak, tired and short of breath, and has problems with | 0.149 |
| infectious diseases |  | activities that require physical effort or deep concentration. | (0.101-0.209) |
|  |  |  |  |
|  |  |  |  |
| Severe anemia due to maternal | Anemia, severe | feels very weak, tired and short of breath, and has problems with | 0.149 |
| hemorrhage |  | activities that require physical effort or deep concentration. | (0.101-0.209) |
| Severe iron-deficiency anemia | Anemia, severe | feels very weak, tired and short of breath, and has problems with | 0.149 |
|  |  | activities that require physical effort or deep concentration. | (0.101-0.209) |
|  |  |  |  |
|  |  |  |  |
| Severe anemia due to peptic | Anemia, severe | feels very weak, tired and short of breath, and has problems with | 0.149 |
| ulcer disease |  | activities that require physical effort or deep concentration. | (0.101-0.209) |
|  |  |  |  |
|  |  |  |  |
| Asymptomatic peptic ulcer | Anemia, severe | feels very weak, tired and short of breath, and has problems with | 0.149 |
| disease with severe anemia |  | activities that require physical effort or deep concentration. | (0.101-0.209) |
|  |  |  |  |
|  |  |  |  |

| Severe anemia due to gastritis | Anemia, severe | feels very weak, tired and short of breath, and has problems with | 0.149 |
| --- | --- | --- | --- |
| and duodenitis |  | activities that require physical effort or deep concentration. | (0.101-0.209) |
|  |  |  |  |
|  |  |  |  |
| Asymptomatic gastritis and | Anemia, severe | feels very weak, tired and short of breath, and has problems with | 0.149 |
| duodenitis with severe anemia |  | activities that require physical effort or deep concentration. | (0.101-0.209) |
|  |  |  |  |
| Stage III chronic kidney |  | feels very weak, tired and short of breath, and has problems with | 0.149 |
| disease and severe anemia due | Anemia, severe |  |  |
|  |  | activities that require physical effort or deep concentration. | (0.101-0.209) |
| to type 1 diabetes mellitus |  |  |  |
|  |  |  |  |
|  |  |  |  |
| Stage III chronic kidney |  | feels very weak, tired and short of breath, and has problems with | 0.149 |
| disease and severe anemia due | Anemia, severe |  |  |
|  |  | activities that require physical effort or deep concentration. | (0.101-0.209) |
| to type 2 diabetes mellitus |  |  |  |
|  |  |  |  |
|  |  |  |  |
| Stage III chronic kidney |  | feels very weak, tired and short of breath, and has problems with | 0.149 |
| disease and severe anemia due | Anemia, severe |  |  |
|  |  | activities that require physical effort or deep concentration. | (0.101-0.209) |
| to hypertension |  |  |  |
|  |  |  |  |
|  |  |  |  |
| Stage III chronic kidney |  | feels very weak, tired and short of breath, and has problems with | 0.149 |
| disease and severe anemia due | Anemia, severe |  |  |
|  |  | activities that require physical effort or deep concentration. | (0.101-0.209) |
| to glomerulonephritis |  |  |  |
|  |  |  |  |
|  |  |  |  |
| Stage III chronic kidney |  | feels very weak, tired and short of breath, and has problems with | 0.149 |
| disease and severe anemia due | Anemia, severe |  |  |
|  |  | activities that require physical effort or deep concentration. | (0.101-0.209) |
| to other causes |  |  |  |
|  |  |  |  |
|  |  |  |  |
| Severe anemia due to | Anemia, severe | feels very weak, tired and short of breath, and has problems with | 0.149 |
| menstrual disorders |  | activities that require physical effort or deep concentration. | (0.101-0.209) |
|  |  |  |  |
|  |  |  |  |
| Severe anemia due to | Anemia, severe | feels very weak, tired and short of breath, and has problems with | 0.149 |
| hemoglobin E trait |  | activities that require physical effort or deep concentration. | (0.101-0.209) |
|  |  |  |  |
|  |  |  |  |
| Severe anemia due to B- | Anemia, severe | feels very weak, tired and short of breath, and has problems with | 0.149 |
| thalassemia trait |  | activities that require physical effort or deep concentration. | (0.101-0.209) |
|  |  |  |  |
|  |  |  |  |
| Severe anemia due to sickle | Anemia, severe | feels very weak, tired and short of breath, and has problems with | 0.149 |
| cell trait |  | activities that require physical effort or deep concentration. | (0.101-0.209) |
|  |  |  |  |
|  |  |  |  |
| Severe anemia due to G6PD | Anemia, severe | feels very weak, tired and short of breath, and has problems with | 0.149 |
| deficiency |  | activities that require physical effort or deep concentration. | (0.101-0.209) |
|  |  |  |  |
|  |  |  |  |
| Severe anemia due to | Anemia, severe | feels very weak, tired and short of breath, and has problems with | 0.149 |
| hemizygous G6PD deficiency |  | activities that require physical effort or deep concentration. | (0.101-0.209) |
|  |  |  |  |
|  |  |  |  |
| Severe anemia due to other |  | feels very weak, tired and short of breath, and has problems with | 0.149 |
| hemoglobinopathies and | Anemia, severe |  |  |
|  |  | activities that require physical effort or deep concentration. | (0.101-0.209) |
| hemolytic anemias |  |  |  |
|  |  |  |  |
|  |  |  |  |
| Severe anemia due to |  | feels very weak, tired and short of breath, and has problems with | 0.149 |
| endocrine, metabolic, blood, | Anemia, severe |  |  |
|  |  | activities that require physical effort or deep concentration. | (0.101-0.209) |
| and immune disorders |  |  |  |
|  |  |  |  |
|  |  |  |  |
|  | Major depressive | feels persistent sadness and has lost interest in usual activities. The | 0.145 |
| Mild major depressive disorder | disorder, mild | person sometimes sleeps badly, feels tired, or has trouble concentrating |  |
|  |  |  | (0.099-0.209) |
|  | episode | but still manages to function in daily life with extra effort. |  |
|  |  |  |  |
|  |  |  |  |
|  | Major depressive | feels persistent sadness and has lost interest in usual activities. The | 0.145 |
| Symptomatic dysthymia | disorder, mild | person sometimes sleeps badly, feels tired, or has trouble concentrating |  |
|  |  |  | (0.099-0.209) |
|  | episode | but still manages to function in daily life with extra effort. |  |
|  |  |  |  |
|  |  |  |  |
| Depression due to premenstrual | Major depressive | feels persistent sadness and has lost interest in usual activities. The | 0.145 |
| syndrome | disorder, mild | person sometimes sleeps badly, feels tired, or has trouble concentrating | (0.099-0.209) |
|  | episode | but still manages to function in daily life with extra effort. |  |
|  |  |  |  |
|  |  |  |  |
| Moderate endocrine, |  |  | 0.145 |
| metabolic, blood, and immune | Hyperthyroidism | feels nervous, has palpitations, sweats a lot and has difficulty sleeping. |  |
|  |  |  | (0.096-0.202) |
| disorders |  |  |  |
|  |  |  |  |
| Incontinence due to | Urinary incontinence | cannot control urinating. | 0.139 |
| encephalocele |  |  | (0.094-0.198) |
|  |  |  |  |
| Incontinence due to congenital | Urinary incontinence | cannot control urinating. | 0.139 |
| anomalies of the urinary tract |  |  | (0.094-0.198) |
|  | Anxiety disorders, | feels anxious and worried, which makes it difficult to concentrate, | 0.133 |
| Moderate anxiety disorders |  | remember things, and sleep. The person tires easily and finds it difficult |  |
|  | moderate |  | (0.091-0.186) |
|  |  | to perform daily activities. |  |
|  |  |  |  |
| Moderate other mental | Anxiety disorders, | feels anxious and worried, which makes it difficult to concentrate, | 0.133 |
|  |  | remember things, and sleep. The person tires easily and finds it difficult |  |
| disorders | moderate |  | (0.091-0.186) |
|  |  | to perform daily activities. |  |
|  |  |  |  |
| Diabetic neuropathy due to | Diabetic neuropathy | has pain, tingling and numbness in the arms, legs, hands and feet. The | 0.133 |
| diabetes mellitus type 1 |  | person sometimes gets cramps and muscle weakness. | (0.089-0.187) |
|  |  |  |  |
| Diabetic neuropathy due to | Diabetic neuropathy | has pain, tingling and numbness in the arms, legs, hands and feet. The | 0.133 |
| diabetes mellitus type 2 |  | person sometimes gets cramps and muscle weakness. | (0.089-0.187) |
|  |  |  |  |

| Severe lower respiratory | Infectious disease, | has a high fever and pain, and feels very weak, which causes great | 0.133 |
| --- | --- | --- | --- |
| infections | acute episode, severe | difficulty with daily activities. | (0.088-0.190) |
|  |  |  |  |
| Severe typhoid fever | Infectious disease, | has a high fever and pain, and feels very weak, which causes great | 0.133 |
|  | acute episode, severe | difficulty with daily activities. | (0.088-0.190) |
|  |  |  |  |
|  |  |  |  |
| Severe paratyphoid fever | Infectious disease, | has a high fever and pain, and feels very weak, which causes great | 0.133 |
|  | acute episode, severe | difficulty with daily activities. | (0.088-0.190) |
|  |  |  |  |
|  |  |  |  |
| Severe acute iNTS | Infectious disease, | has a high fever and pain, and feels very weak, which causes great | 0.133 |
|  | acute episode, severe | difficulty with daily activities. | (0.088-0.190) |
|  |  |  |  |
|  |  |  |  |
| Severe malaria | Infectious disease, | has a high fever and pain, and feels very weak, which causes great | 0.133 |
|  | acute episode, severe | difficulty with daily activities. | (0.088-0.190) |
|  |  |  |  |
|  |  |  |  |
| Severe visceral leishmaniasis | Infectious disease, | has a high fever and pain, and feels very weak, which causes great | 0.133 |
|  | acute episode, severe | difficulty with daily activities. | (0.088-0.190) |
|  |  |  |  |
|  |  |  |  |
| Severe dengue | Infectious disease, | has a high fever and pain, and feels very weak, which causes great | 0.133 |
|  | acute episode, severe | difficulty with daily activities. | (0.088-0.190) |
|  |  |  |  |
|  |  |  |  |
| Severe yellow fever | Infectious disease, | has a high fever and pain, and feels very weak, which causes great | 0.133 |
|  | acute episode, severe | difficulty with daily activities. | (0.088-0.190) |
|  |  |  |  |
|  |  |  |  |
| Rabies | Infectious disease, | has a high fever and pain, and feels very weak, which causes great | 0.133 |
|  | acute episode, severe | difficulty with daily activities. | (0.088-0.190) |
|  |  |  |  |
|  |  |  |  |
| Ebola cases | Infectious disease, | has a high fever and pain, and feels very weak, which causes great | 0.133 |
|  | acute episode, severe | difficulty with daily activities. | (0.088-0.190) |
|  |  |  |  |
|  |  |  |  |
| Acute pneumococcal | Infectious disease, | has a high fever and pain, and feels very weak, which causes great | 0.133 |
| meningitis | acute episode, severe | difficulty with daily activities. | (0.088-0.190) |
|  |  |  |  |
| Acute H influenzae type B | Infectious disease, | has a high fever and pain, and feels very weak, which causes great | 0.133 |
| meningitis | acute episode, severe | difficulty with daily activities. | (0.088-0.190) |
|  |  |  |  |
| Acute meningococcal | Infectious disease, | has a high fever and pain, and feels very weak, which causes great | 0.133 |
| meningitis | acute episode, severe | difficulty with daily activities. | (0.088-0.190) |
|  |  |  |  |
| Other acute bacterial | Infectious disease, | has a high fever and pain, and feels very weak, which causes great | 0.133 |
| meningitis | acute episode, severe | difficulty with daily activities. | (0.088-0.190) |
|  |  |  |  |
| Acute viral meningitis | Infectious disease, | has a high fever and pain, and feels very weak, which causes great | 0.133 |
|  | acute episode, severe | difficulty with daily activities. | (0.088-0.190) |
|  |  |  |  |
|  |  |  |  |
| Acute encephalitis | Infectious disease, | has a high fever and pain, and feels very weak, which causes great | 0.133 |
|  | acute episode, severe | difficulty with daily activities. | (0.088-0.190) |
|  |  |  |  |
|  |  |  |  |
| Severe diphtheria | Infectious disease, | has a high fever and pain, and feels very weak, which causes great | 0.133 |
|  | acute episode, severe | difficulty with daily activities. | (0.088-0.190) |
|  |  |  |  |
|  |  |  |  |
| Severe tetanus | Infectious disease, | has a high fever and pain, and feels very weak, which causes great | 0.133 |
|  | acute episode, severe | difficulty with daily activities. | (0.088-0.190) |
|  |  |  |  |
|  |  |  |  |
| Severe measles | Infectious disease, | has a high fever and pain, and feels very weak, which causes great | 0.133 |
|  | acute episode, severe | difficulty with daily activities. | (0.088-0.190) |
|  |  |  |  |
|  |  |  |  |
| Severe acute hepatitis A | Infectious disease, | has a high fever and pain, and feels very weak, which causes great | 0.133 |
|  | acute episode, severe | difficulty with daily activities. | (0.088-0.190) |
|  |  |  |  |
|  |  |  |  |
| Severe acute hepatitis B | Infectious disease, | has a high fever and pain, and feels very weak, which causes great | 0.133 |
|  | acute episode, severe | difficulty with daily activities. | (0.088-0.190) |
|  |  |  |  |
|  |  |  |  |
| Severe acute hepatitis C | Infectious disease, | has a high fever and pain, and feels very weak, which causes great | 0.133 |
|  | acute episode, severe | difficulty with daily activities. | (0.088-0.190) |
|  |  |  |  |
|  |  |  |  |
| Severe acute hepatitis E | Infectious disease, | has a high fever and pain, and feels very weak, which causes great | 0.133 |
|  | acute episode, severe | difficulty with daily activities. | (0.088-0.190) |
|  |  |  |  |
|  |  |  |  |
| Puerperal sepsis | Infectious disease, | has a high fever and pain, and feels very weak, which causes great | 0.133 |
|  | acute episode, severe | difficulty with daily activities. | (0.088-0.190) |
|  |  |  |  |
|  |  |  |  |
| Severe infection due to | Infectious disease, | has a high fever and pain, and feels very weak, which causes great | 0.133 |
| neonatal sepsis and other |  |  |  |
|  | acute episode, severe | difficulty with daily activities. | (0.088-0.190) |
| neonatal infections |  |  |  |
|  |  |  |  |
| Severe endocarditis | Infectious disease, | has a high fever and pain, and feels very weak, which causes great | 0.133 |
|  | acute episode, severe | difficulty with daily activities. | (0.088-0.190) |
|  |  |  |  |
|  |  |  |  |
| Severe cellulitis | Infectious disease, | has a high fever and pain, and feels very weak, which causes great | 0.133 |
|  | acute episode, severe | difficulty with daily activities. | (0.088-0.190) |
|  |  |  |  |
|  |  |  |  |
|  |  | has wheezing, cough and shortness of breath more than twice a week, | 0.133 |
| Uncontrolled asthma | Asthma, uncontrolled | which causes difficulty with daily activities and sometimes wakes the |  |
|  |  |  | (0.086-0.192) |
|  |  | person at night. |  |
|  |  |  |  |

| Epididymo-orchitis due to | Epididymo-orchitis | has swelling and tenderness in the testicles and pain during urination. | 0.128 |
| --- | --- | --- | --- |
| chlamydial infection |  |  | (0.086-0.180) |
|  |  |  |  |
| Epididymo-orchitis due to | Epididymo-orchitis | has swelling and tenderness in the testicles and pain during urination. | 0.128 |
| gonococcal infection |  |  | (0.086-0.180) |
|  |  |  |  |
| Hydrocele due to lymphatic | Epididymo-orchitis | has swelling and tenderness in the testicles and pain during urination. | 0.128 |
| filariasis |  |  | (0.086-0.180) |
|  |  |  |  |
| Severe wasting due to | Severe wasting | is extremely skinny and has no energy. | 0.128 |
| ascariasis |  |  | (0.082-0.183) |
|  |  |  |  |
| Severe wasting due to | Severe wasting | is extremely skinny and has no energy. | 0.128 |
| trichuriasis |  |  | (0.082-0.183) |
|  |  |  |  |
| Severe wasting due to | Severe wasting | is extremely skinny and has no energy. | 0.128 |
| hookworm disease |  |  | (0.082-0.183) |
|  |  |  |  |
| Severe wasting without edema | Severe wasting | is extremely skinny and has no energy. | 0.128 |
|  |  |  | (0.082-0.183) |
|  |  |  |  |
| Very mild alcohol dependence | Alcohol use disorder, | drinks alcohol daily and has difficulty controlling the urge to drink. | 0.123 |
|  | very mild | When sober, the person functions normally. | (0.082-0.177) |
|  |  |  |  |
