# Supplementary material for: Revised estimates of leprosy disability weights for assessing the global burden of disease: A systematic review and individual patient data meta-analysis
Source: PLoS Negl Trop Dis. 2021 Mar 2;15(3):e0009209. doi: 10.1371/journal.pntd.0009209 (PMC7954345; doi:10.1371/journal.pntd.0009209)
Supplement: S1 Appendix — (DOCX) [file pntd.0009209.s003.docx]

S1 Appendix

Search strategy and selection criteria

**Table A. Summary of search results**

| *Databases* | *References* | *After de-duplication* |
| --- | --- | --- |
| Embase.com | 1316 | 1293 |
| Medline (Ovid) | 1174 | 521 |
| Web of Science | 794 | 261 |
| Cochrane CENTRAL | 32 | 6 |
| Google Scholar *top 200 relevant references* | 200 | 114 |
| **Total** | **3516** | **2195** |

**Embase.com**

('leprosy'/exp OR 'Mycobacterium leprae'/de OR 'leprosy epidemiology'/de OR ('neglected disease'/de AND 'tropical disease'/de) OR (lepros* OR (Hansen NEXT/2 disease*) OR lepra* OR (neglect* NEAR/3 tropical* NEAR/3 disease*)):ab,ti) AND ('disease burden'/de OR 'quality adjusted life year'/de OR 'economic evaluation'/exp OR 'quality of life assessment'/exp OR 'quality of life'/exp OR (((qualit*) NEAR/3 (life-year* OR lifeyear*)) OR QALY* OR economic* OR ((cost*) NEAR/3 (util* OR benefi* OR effectiv*)) OR ((euroqol OR euro-qol OR euroquol OR euro-quol) NEAR/3 (3 OR 3D OR three OR 5 OR 5D OR five)) OR EQ-3D OR EQ3D OR EQOL-3D OR EQOL3D OR EQ-5D OR EQ5D OR EQOL-5D OR EQOL5D OR EuroQol3 OR EuroQol5 OR QOL OR HRQOL OR HRQL OR (qualit* NEAR/3 life*) OR WHOqol*):ab,ti) NOT ([animals]/lim NOT [humans]/lim)

**Medline (Ovid)**

(exp Leprosy/ OR Mycobacterium leprae/ OR Neglected Diseases/ OR (neglected disease/ AND tropical disease/) OR (lepros* OR (Hansen ADJ2 disease*) OR lepra* OR (neglect* ADJ3 tropical* ADJ3 disease*)).ab,ti.) AND (exp Health Status Indicators/ OR exp "Costs and Cost Analysis"/ OR Quality of Life/ OR (((qualit*) ADJ3 (life-year* OR lifeyear*)) OR QALY* OR economic* OR ((cost*) ADJ3 (util* OR benefi* OR effectiv*)) OR ((euroqol OR euro-qol OR euroquol OR euro-quol) ADJ3 (3 OR 3D OR three OR 5 OR 5D OR five)) OR EQ-3D OR EQ3D OR EQOL-3D OR EQOL3D OR EQ-5D OR EQ5D OR EQOL-5D OR EQOL5D OR EuroQol3 OR EuroQol5 OR QOL OR HRQOL OR HRQL OR (qualit* ADJ3 life*) OR WHOqol*).ab,ti.) NOT (exp animals/ NOT humans/)

**Web of Science**

TS=(((lepros* OR (Hansen NEAR/2 disease*) OR lepra* OR (neglect* NEAR/2 tropical* NEAR/2 disease*))) AND ((((qualit*) NEAR/2 (life-year* OR lifeyear*)) OR QALY* OR economic* OR ((cost*) NEAR/2 (util* OR benefi* OR effectiv*)) OR ((euroqol OR euro-qol OR euroquol OR euro-quol) NEAR/2 (3 OR 3D OR three OR 5 OR 5D OR five)) OR EQ-3D OR EQ3D OR EQOL-3D OR EQOL3D OR EQ-5D OR EQ5D OR EQOL-5D OR EQOL5D OR EuroQol3 OR EuroQol5 OR QOL OR HRQOL OR HRQL OR (qualit* NEAR/2 life*) OR WHOqol*)) NOT ((animal* OR rat OR rats OR mouse OR mice OR murine OR dog OR dogs OR canine OR cat OR cats OR feline OR rabbit OR cow OR cows OR bovine OR rodent* OR sheep OR ovine OR pig OR swine OR porcine OR veterinar* OR chick* OR zebrafish* OR baboon* OR nonhuman* OR primate* OR cattle* OR goose OR geese OR duck OR macaque* OR avian* OR bird* OR fish*) NOT (human* OR patient* OR women OR woman OR men OR man)))

**Cochrane CENTRAL**

((lepros* OR (Hansen NEXT/2 disease*) OR lepra* OR (neglect* NEAR/3 tropical* NEAR/3 disease*)):ab,ti) AND ((((qualit*) NEAR/3 (life-year* OR lifeyear*)) OR QALY* OR economic* OR ((cost*) NEAR/3 (util* OR benefi* OR effectiv*)) OR ((euroqol OR euro-qol OR euroquol OR euro-quol) NEAR/3 (3 OR 3D OR three OR 5 OR 5D OR five)) OR EQ-3D OR EQ3D OR EQOL-3D OR EQOL3D OR EQ-5D OR EQ5D OR EQOL-5D OR EQOL5D OR EuroQol3 OR EuroQol5 OR QOL OR HRQOL OR HRQL OR (qualit* NEAR/3 life*) OR WHOqol*):ab,ti)

**Google Scholar** *top 200 relevant references*

leprosy|leprosum|"Hansen disease"|lepra|leprosis "quality adjusted life year"|QALY|"cost utility|benefit|effectiveness"|EQ-3D|EQ-5D|EuroQol|"quality of life"

**Selection Criteria:**

*(The eligibility criteria were formulated using PICOS guidelines)*

Inclusion Criteria:

- Studies conducted on human populations that have been affected, treated or cured of Leprosy, globally.
- Studies mentioning and providing any Quality of Life estimates on leprosy, irrespective of the type of instrument/tool used for assessing the same.
- Eligible studies for the review will include: a.) Analytical study designs (cohort studies, case-control studies, analytical cross-sectional studies) and, b.) Descriptive study designs (cross-sectional studies), and c.) Experimental studies.

Exclusion criteria:

- Studies that have no association with Quality of life in leprosy patients.
- Studies before 1990
- The review will include all study designs except for case reports, and case series.
- Studies that do not provide any quantitative estimates for quality of life in the results or have aggregated results (with and without leprosy aggregated together).
- Studies for whom full-text articles and abstract are unavailable or inaccessible.
- Studies will be limited to human populations. All animal related studies will be excluded

All published and unpublished studies will be included and there will be no restriction in language or type of geographical setting.
