# Supplementary material for: Revised estimates of leprosy disability weights for assessing the global burden of disease: A systematic review and individual patient data meta-analysis
Source: PLoS Negl Trop Dis. 2021 Mar 2;15(3):e0009209. doi: 10.1371/journal.pntd.0009209 (PMC7954345; doi:10.1371/journal.pntd.0009209)
Supplement: S2 Appendix — (DOCX) [file pntd.0009209.s004.docx]

S2 Appendix.

Summary of missing data, multiple imputation and disability weight transformation

R Package: mice

Number of imputations: m= 5

Number of iterations: maxit= 30

**Table A. Missingness in the study datasets**

| **Name of study** | **Missing variable** | **No. of missing values** | **Variable Type** | **Imputation method** | **Number of predictor variables** |
| --- | --- | --- | --- | --- | --- |
| Guimenes, et al., 2019 | Disability Grade | 43 | Ordered factor with 3 levels (0,1,2) | polr | 11 |
| Lustosa, et al., 2011 | Disability Grade | 5 | Ordered factor with 3 levels (0,1,2) | polr | 11 |
| Bowers, et al., 2017 | All 8 HRQOL domains | 1 | Numeric | pmm | 11 |
| Lambert, et al., 2016 (Trial 2) | Social Functioning (SF) domain | 1 | Numeric | pmm | 11 |
| Watanabe, H., 2013 | Type of Leprosy | **143** | **NI** | **NI** | **NI** |

*pmm- predictive mean matching; polr- proportional odds model; NI- Not Imputed (because variable not required for analyses)*

Convergence plots

Fig A. Guimenes, et al., 2019


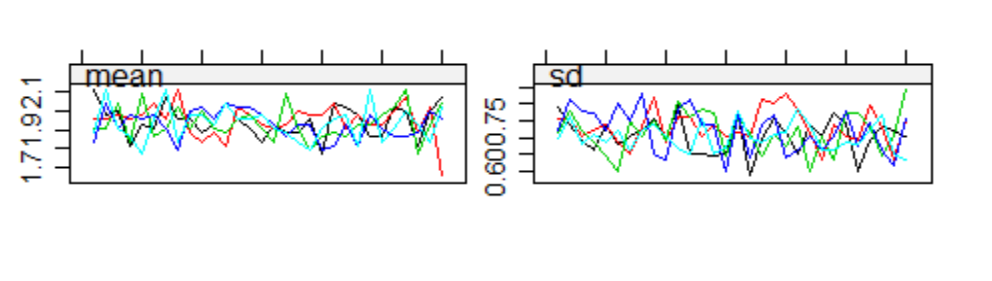


Fig B. Lustosa, et al., 2011


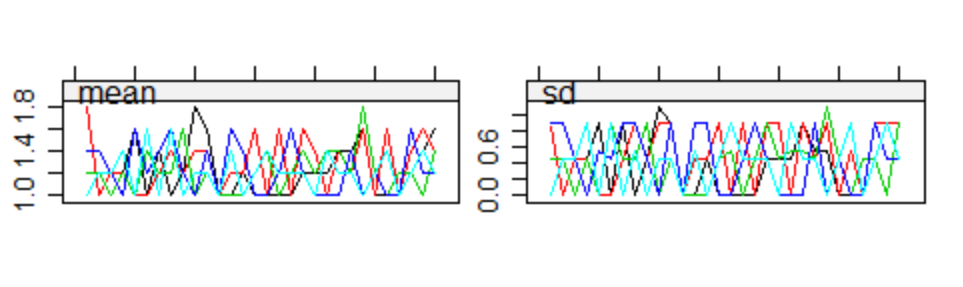


Fig C. Bowers, et al., 2017


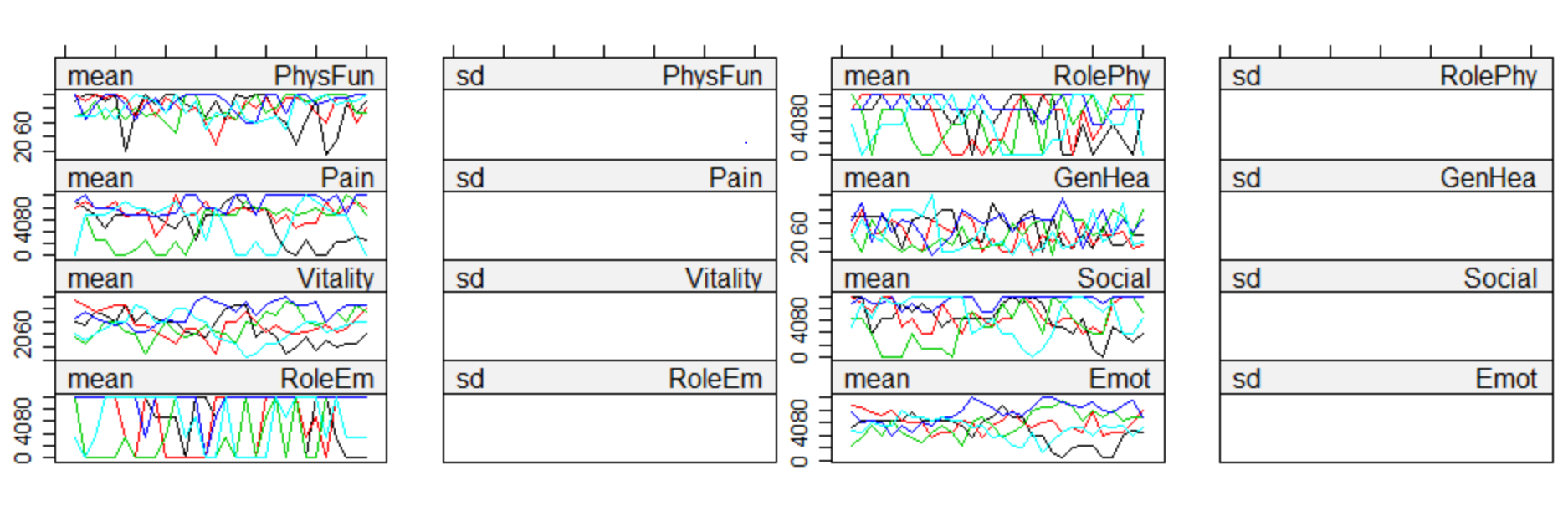


Fig D. Lambert, et al., 2016 (Trial 2)


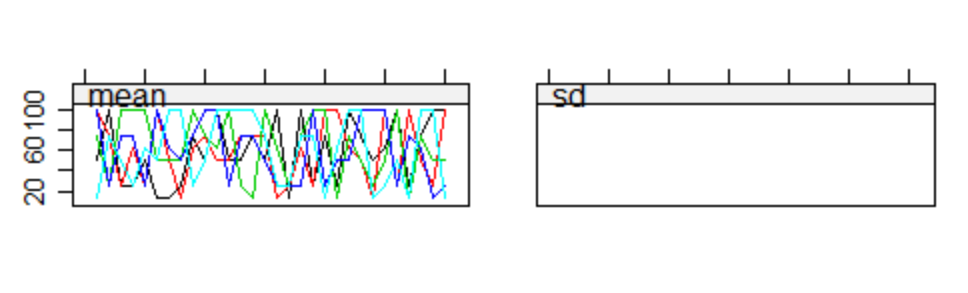


**Table B. Summary of SF score to disability weight transformation**

| **Range of SF-Score** | **Result** |
| --- | --- |
| 59.6 – 105.6 | Loess function returned a disability weight for any scores within this range |
| > 116.34 | Based on truncated function by Burstein et al., score above this level was set to 0 |
| < 43.0 | Based on truncated function by Burstein et al., score below this level was set to 1 |
| 43.1 – 59.5  &  105.7 – 116.33 | Constant slopes assumed, and disability weight calculated for each SF-score after calculation of slopes (m1,m2) and by using the formulas: -m1*(SF-Score-43)+1;  -m2*(SF-Score-105.6)+0.0081 |

**Table C. Sensitivity analyses of multiple imputation- Study results**

| **Sensitivity analyses** | **Guimenes, et al., 2019**  (43 missing grade values) | | | **Lustosa, et al., 2011**  (5 missing grade values) | | | **Bowers, et al., 2017** (1 observ. missing- all 8 domain scores) | **Lambert, et al., 2016 (Trial 2)** (1 observ. missing-1 domain score) |
| --- | --- | --- | --- | --- | --- | --- | --- | --- |
|  | Mean DW [95% CI] | | | Mean DW [95% CI] | | | Mean DW  [95% CI] | Mean DW  [95% CI] |
|  | G0 | G1 | G2 | G0 | G1 | G2 | G0 | G2 |
| With Imputation | **0.05** [-0.01-0.11] | **0.11** [0.06-0.17] | **0.21** [0.07-0.34] | **0.03** [0.01-0.04] | **0.11** [0.03-0.15] | **0.18** [0.03-0.33] | **0.19**  [0.09-0.29] | **0.18**  [0.08-0.27] |
| Without Imputation | **0.07**  [-0.02-0.16] | **0.12**  [0.06-0.18] | **0.23**  [0.07-0.39] | **0.03**  [0.01-0.05] | **0.09**  [0.03-0.15] | **0.19**  [0.04-0.34] | **0.19**  [0.08-0.30] | **0.14**  [0.08-0.20] |

*G0- Grade 0, G1- Grade 1, G2- Grade 2, DW- Disability weights, observ.- observation, CI- Confidence interval*

**Table D. Sensitivity analyses of multiple imputation- Overall results**

|  | **Multivariate Meta-Analyzed Mean Disability Weights [95% CI]** | | |
| --- | --- | --- | --- |
|  | **Grade 0** | **Grade 1** | **Grade 2** |
| With Imputation | **0.12**  [0.06- 0.19] | **0.19**  [0.13- 0.26] | **0.26**  [0.18- 0.34] |
| Without Imputation | **0.14**  [0.08-0.20] | **0.20**  [0.13-0.26] | **0.27**  [0.20-0.35] |

*CI- Confidence Interval*
