# Supplementary material for: Revised estimates of leprosy disability weights for assessing the global burden of disease: A systematic review and individual patient data meta-analysis
Source: PLoS Negl Trop Dis. 2021 Mar 2;15(3):e0009209. doi: 10.1371/journal.pntd.0009209 (PMC7954345; doi:10.1371/journal.pntd.0009209)
Supplement: S3 Appendix — (DOCX) [file pntd.0009209.s005.docx]

S3 Appendix.

Summary of Meta-analyzed domain scores

**Table A. Difference between grades of disability**

|  | Disability Weight Estimate | Standard Error | 95% Confidence Interval | | | zval | Pval |
| --- | --- | --- | --- | --- | --- | --- | --- |
|  |  |  | **Lower** | | **Upper** |  |  |
| intercept | **0.12** | 0.03 | 0.06 | 0.18 | | 4.02 | <0.0001 |
| Grade 1 | **0.07** | 0.02 | 0.13 | 0.26 | | 3.32 | 0.0009 * |
| Grade 2 | **0.14** | 0.05 | 0.18 | 0.34 | | 3.04 | 0.0024 * |

Zval- Z value; pval- p value; reference grade- Grade 0

* statistically significant (p < 0.05)

**Table B. Multivariate meta-analysis of domain scores by grade (8 studies with IPD)**

|  | PF | RP | BP | GH |
| --- | --- | --- | --- | --- |
| Grade 0 | 83.94  [79.54-88.35] | 63.57  [52.57-74.57] | 54.39  [42.17-66.6] | 52.58  [44.2-60.95] |
| Grade 1 | `69.85  [62.77-76.92] | 53.24  [39.73-66.76] | 46.5  [35.27-57.73] | 43.70  [35.17-52.24] |
| Grade 2 | 55.99  [47.02-64.97 | 37.74  [28.57-46.9] | 42.18  [31.48-52.88] | 42.05  [33.34-50.75] |
|  | **VT** | **SF** | **RE** | **MH** |
| Grade 0 | 59.31  [50.39-68.23] | 83.04  [77.27-88.82] | 69.05  [54.62-83.48] | 58.51  [49.77-67.24] |
| Grade 1 | 51.54  [43.76-59.31] | 76.83  [71.27-82.38] | 56.51  [42.60-70.42] | 53.16  [44.51-61.8] |
| Grade 2 | 50.06  [43.19-56.93] | 63.84  [57.01-70.66] | 53  [42.78-63.21] | 55.03  [48.16-61.9] |

*PF- Physical Function, RP- Role limitations (physical), BP- Bodily Pain, GH- General Health, VT- Vitality, SF- Social Functioning, RE- Role limitations (emotional), MH- Mental Health*

**Table C. Results of multivariate random effects meta-analysis of domain scores by grade**

Physical Function (PF)

estimate se zval pval ci.lb ci.ub

intrcpt 83.9437 2.2480 37.3418 <.0001 79.5378 88.3497 ***

grade1 -14.0973 3.1148 -4.5259 <.0001 -20.2021 -7.9924 ***

grade2 -27.9510 4.5094 -6.1984 <.0001 -36.7893 -19.1128 ***

Role limitations (physical) (RP)

estimate se zval pval ci.lb ci.ub

intrcpt 63.5661 5.6118 11.3272 <.0001 52.5671 74.5651 ***

grade1 -10.3239 10.5952 -0.9744 0.3299 -31.0902 10.4423

grade2 -25.8299 5.0829 -5.0817 <.0001 -35.7923 -15.8676 ***

Bodily Pain (BP)

estimate se zval pval ci.lb ci.ub

intrcpt 54.3854 6.2338 8.7243 <.0001 42.1674 66.6035 ***

grade1 -7.8840 3.0240 -2.6071 0.0091 -13.8109 -1.9570 **

grade2 -12.2069 3.6853 -3.3123 0.0009 -19.4299 -4.9838 ***

General Health (GH)

estimate se zval pval ci.lb ci.ub

intrcpt 52.5761 4.2742 12.3009 <.0001 44.1989 60.9534 ***

grade1 -8.8714 2.5428 -3.4889 0.0005 -13.8551 -3.8877 ***

grade2 -10.5304 2.8782 -3.6587 0.0003 -16.1715 -4.8893 ***

Vitality (VT)

estimate se zval pval ci.lb ci.ub

intrcpt 59.3080 4.5510 13.0317 <.0001 50.3881 68.2278 ***

grade1 -7.7716 2.4531 -3.1681 0.0015 -12.5796 -2.9636 **

grade2 -9.2458 2.9089 -3.1784 0.0015 -14.9472 -3.5443 **

Social Functioning (SF)

estimate se zval pval ci.lb ci.ub

intrcpt 83.0438 2.9470 28.1788 <.0001 77.2677 88.8198 ***

grade1 -6.2176 3.1060 -2.0018 0.0453 -12.3053 -0.1298 *

grade2 -19.2060 5.0094 -3.8340 0.0001 -29.0242 -9.3878 ***

Role limitations (emotional) (RE)

estimate se zval pval ci.lb ci.ub

intrcpt 69.0475 7.3625 9.3783 <.0001 54.6173 83.4778 ***

grade1 -12.5345 4.9881 -2.5129 0.0120 -22.3110 -2.7580 *

grade2 -16.0521 9.3956 -1.7085 0.0875 -34.4671 2.3629 .

Mental Health (MH)

estimate se zval pval ci.lb ci.ub

intrcpt 58.5083 4.4566 13.1284 <.0001 49.7735 67.2430 ***

grade1 -5.3495 2.4840 -2.1535 0.0313 -10.2181 -0.4808 *

grade2 -3.4794 3.3922 -1.0257 0.3050 -10.1280 3.1692

*se- Standard error; zval- Z value; pval- P value; ci.lb- Lower bounds of 95% Confidence interval;*

*ci.ub- Upper bounds of 95% Confidence interval; intrcpt- Intercept (reference grade-0)*

**Table D. Multivariate meta-analysis of domain scores of all 14 studies**

| Domain | Domain Mean Estimate | Standard Error | 95% Confidence Interval | | |
| --- | --- | --- | --- | --- | --- |
|  |  |  | **Lower** | | **Upper** |
| Physical Functioning -PF | 60.0063 | 3.2921 | 53.5540 | 66.4586 | |
| Role limitations- RP (physical) | 41.4173 | 3.6211 | 34.3200 | 48.5146 | |
| Bodily Pain- BP | 50.0993 | 3.6614 | 42.9231 | 57.2756 | |
| General Health- GH | 50.3201 | 2.9769 | 44.4856 | 56.1547 | |
| Vitality- VT | 54.7327 | 2.8780 | 49.0919 | 60.3735 | |
| Social Functioning- SF | 66.3792 | 2.5586 | 61.3643 | 71.3940 | |
| Role limitations -RE (emotional) | 50.8895 | 4.1127 | 42.8288 | 58.9503 | |
| Mental Health- MH | 57.4886 | 2.7063 | 52.1843 | 62.7929 | |
