# Supplementary material for: Revised estimates of leprosy disability weights for assessing the global burden of disease: A systematic review and individual patient data meta-analysis
Source: PLoS Negl Trop Dis. 2021 Mar 2;15(3):e0009209. doi: 10.1371/journal.pntd.0009209 (PMC7954345; doi:10.1371/journal.pntd.0009209)
Supplement: Attachment — Submitted filename: List of responses.docx [file pntd.0009209.s006.docx]

**RESPONSES TO PLOS NTD REVIEWER COMMENTS**

Methods

-Are the objectives of the study clearly articulated with a clear testable hypothesis stated?

-Is the study design appropriate to address the stated objectives?

-Is the population clearly described and appropriate for the hypothesis being tested?

-Is the sample size sufficient to ensure adequate power to address the hypothesis being tested?

-Were correct statistical analysis used to support conclusions?

-Are there concerns about ethical or regulatory requirements being met?

Reviewer #1: Yes to all questions above

Reviewer #2: This is a well defined and organized study

Results
-Does the analysis presented match the analysis plan?

-Are the results clearly and completely presented?

-Are the figures (Tables, Images) of sufficient quality for clarity?

Reviewer #1: Table 1: No explanation is given for the acronym 'RAND-36' or its equivalence with the SF-36

**Response**: Expansion of RAND- 'Research and Development' and SF- 'Short Form', has been added in the footnotes of Table 1, along with a brief summary of the RAND-36.

In addition, ‘any version’ has been added in Line 161, to indicate that there are multiple versions of the SF-36 in use.

Table 5: People with Grade 1 disabilities also face participation restrictions and suffer from depression. Grade 2 disability is a frequent cause of depression and of severe participation restrictions. A recent systematic review found that up to 50% of persons affected by leprosy suffer from depression and/or anxiety. This is insufficiently captured in the proposed health state descriptions.

**Response:** The term ‘mental distress’ has now been added to the health state descriptions.

Since GBD lay descriptions should contain simple terminologies, we decided not to use terms like ‘depression’ as it should meet more defined diagnostic criteria.

Since SF-36 was designed to provide only a general measure of mental health, upon which we have based our conclusions, we have decided to use ‘mental distress’. Firstly, the contents of the ‘Mental Health’ domain in the SF-36, surveys the experiences of the patients over only a short period of time (past 4 weeks).

Secondly, mental distress is experienced in patients with anxiety disorders, depressive disorders, and is linked with most types of mental disorders.

Reviewer #2: Results are clear and well analyzed

Conclusions
-Are the conclusions supported by the data presented?

-Are the limitations of analysis clearly described?

-Do the authors discuss how these data can be helpful to advance our understanding of the topic under study?

-Is public health relevance addressed?

Reviewer #1: Line 358-65: the authors are advised to consider rephrasing the proposed health state descriptions since 'daily activities' is often taken to mean activities of daily living, i.e. the activity component of the ICF, rather than the participation component. Furthermore, an explicit reference to poor mental wellbeing (or mental health) is recommended.

**Response:** For the purpose of closeness, the terminologies used for our new health descriptions has been restricted to those utilized for other lay descriptions, by IHME. Moreover, the idea was to capture impairment, activity limitation and participation restriction in our new disability descriptions.

Since there is considerable overlap between activity (in a daily situation) and participation (in a life situation), we decided to retain the term ‘daily activities’ to include both these components, and also added the term ‘social activity’ to underscore participation.

As ‘mental health’ is too broad a term and has been avoided by IHME to use in any of the other GBD health descriptions, we have rephrased the description with the term ‘mental distress’ as justified in the previous response.

| **Sequela** | **Newly proposed health state description for leprosy** |
| --- | --- |
| Disfigurement Level 1 due to leprosy  (WHO leprosy grade 0) | Has no visible deformity, but skin lesions and mild pain, which causes some mental distress, but no difficulty with daily activities |
| Disfigurement Level 2 due to leprosy  (WHO leprosy grade 1) | Has a slight visible deformity, often with moderate pain, which often causes some mental distress and some difficulty with social and daily activities |
| Disfigurement Level 3 due to leprosy  (WHO leprosy grade 2) | Has visible deformity, often with severe pain, which often causes a lot of mental distress and great difficulty with social and daily activities |

Line 359, 364- The term ’physical limitation’ is changed to ‘activity limitation’ for uniformity and clarity.

Reviewer #2: The conclusions are logical and supported by the analysis

Editorial and Data Presentation Modifications

Use  this section for editorial suggestions as well as relatively minor modifications of existing data that would enhance clarity. If the only modifications needed are minor and/or editorial, you may wish to recommend "Minor Revision" or "Accept".

Reviewer #1: Discussion
I miss a discussion of the impact of the number of people counted in the DALY calculation (based on new cases only?) and the duration of the disability (only counted during MDT?). If these are not included appropriately, this will also significantly affect the resulting estimates.

**Response**: We did not discuss DALY calculation in leprosy, as the complete methodology include components like conversion of incidence data into prevalence, comorbidity correction, etc., to name a few. Discussions on these goes beyond the scope of this paper, which is to address only the low disability weights and the inaccurate health descriptions, based on the most affected domains. We believe discussing further about other variables that will impact the leprosy DALY estimations would be superfluous information to the reader, and take the focus off the aim of our research.

In addition, though incidence is the measure of choice in the calculation of DALY estimates in the GBD model, the supplementary material (from GBD 2017) does not provide clear or detailed information on the steps and methodology used to obtain the same from prevalence data. We would also like to mention here that we attempted to reach out to IHME scientists on multiple occasions to gather information on the same but unfortunately could not get in touch.

Line 313-4: This may indicate that the SF-36 is not the most appropriate instrument to quantify the experience of depression and anxiety, which are found to be very common among persons affected by leprosy. It would be appropriate to note this in the light of the findings in the below two studies.

Line 316-8: see also Somar et al (2020). The impact of leprosy on the mental wellbeing of leprosy-affected persons and their family members - a systematic review.
Also Van Dorst et al (2020): ref 3 and
Bow-Bertrand et al (2019). An exploration into the psychological impact of leprosy in Sirajganj, Bangladesh

**Response**: We agree that other tools such as the GHQ-12, Beck’s inventory, etc (Somar et al). are more sensitive and produce more accurate estimates for depression and mental health. Though our review did find many studies using these tools, we could not include them as there are currently no valid algorithms or models designed, for converting these scores into GBD disability weights.

We have included the same as a limitation.

**Added in edited manuscript as line 308-312**: *“**Though, the SF-36 has been designed to provide only a general measure of mental health for most conditions, the use of more sensitive instruments for measuring patient experiences like anxiety and depression (Somar et al) would have contributed to more accurate estimates of disability burden in leprosy’*

Line 332: It may be worth noting that this has been found also for other NTDs, e.g.
Bailey F, Mondragon-Shem K, Haines LR, Olabi A, Alorfi A, Ruiz-Postigo JA, Alvar J, Hotez P, Adams ER, Vélez ID, Al-Salem W. Cutaneous leishmaniasis and co-morbid major depressive disorder: A systematic review with burden estimates. PLoS neglected tropical diseases. 2019 Feb 25;13(2):e0007092.
Bailey F, Eaton J, Jidda M, van Brakel WH, Addiss DG, Molyneux DH. Neglected tropical diseases and mental health: Progress, partnerships, and integration. Trends in parasitology. 2019 Jan 1;35(1):23-31.

**Response**: The same has been added in the discussion section along with the citations.

**LINE 337**: (Bailey, Eaton, et al.)- **citation has been included**

**Added to manuscript Line 337-338**: *Modification of disability weights for other NTDs, through the incorporation of the psychological impact of disease has been made in previous GBD studies (Bailey, Mondragon, et al).*

Line 343: I would challenge this statement. HRQOL based weights may do a better job than the previous methods, but the SF-36 may still underestimate the impact, especially on social and work participation and on mental health, since it has not been designed to measure these based on the ICF and DSM frameworks. The very common impact on family members is not taken into account either.

**Response**: As mentioned in a previous response, we have included the same as a limitation. However, we also need to mention that the impact on family members is not taken into account in lay descriptions of any diseases/ injuries, in the GBD studies.

In addition we have rephrased line 350 (in edited manuscript)

**from**- *‘The advantage of HRQOL based weights is that they depict the real burden by covering the patient's perspective’*

**to- *‘****The advantage of HRQOL based weights is that they can sufficiently capture disease burden by covering the patient's perspective’*

Reviewer #2: Probably has too many tables and seems overly technical

**Response**: We have decided to move Table 3- which shows the differences between the grades of disability to the supplementary section, as Table A in S4 Appendix.

Summary and General Comments

Use  this section to provide overall comments, discuss strengths/weaknesses of the study, novelty, significance, general execution and scholarship. You may also include additional comments for the author, including concerns about dual publication, research ethics, or publication ethics. If requesting major revision, please articulate the new experiments that are needed.

Reviewer #1: The DALY weights for leprosy are an important topic since revision has been overdue for a long time!

**Response**: Thank you for your time and comments. We hope this study will serve as a basis for discussion and further changes to leprosy disability weights and burden, in future GBD study editions.

Reviewer #2: The authors present here a meta-analysis of leprosy disability weighting estimates, with particular emphasis on patient input.  They suggest that leprosy is among the more "neglected of the neglected tropical diseases" in that the current antiquated disability estimates erroneously suggest a false low burden of disease in most populations, that can incorrectly skew distribution of health resources or bely the benefits of new intervention programs.  This results mainly from a lack of patient input on psychological and social issues and a traditional reliance of physical impairment scores.

It is mind boggling that a disease so widely known, feared and stigmatized around the world, would not somehow include patient input on mental health and social impacts of their disease.  Yet, among the more than 2000 manuscripts reviewed in this analysis only 14 included standardized patient input and among those only 8 also had robust enough data to be re-analyzed in this study.  Perhaps not surprisingly, they observe that individuals with grade 0 and 1 disabilities also suffer mental health and social deficiencies and the resulting true burden of those disabilities are 4-20 times greater than previously understood.

This is an important contribution to bring leprosy more into the modern world.  Based on solid statistical analysis the authors responsibly offer new disability definitions that can change the paradigm of disability estimation and disease burden in leprosy.  The language of the paper is somewhat technical and the total number of tables could easily be halved.

**Response**: Thank you for your time and comments. Revision of the burden estimates will significantly improve the priority ranking of leprosy among NTDs, and we believe this paper will highlight this when published.

As per your previous comments, we have moved one of the tables (Table 3) to the supplementary section. Due to the need for comparison with previous GBD estimates and descriptions, we have retained the other tables in the manuscript as it will help highlight these comparisons to the reader.

In revision the authors should consider:

Line 222: "a" few
Lines 346-349: poorly phrased.  As written implies the revised weights are not different from before.  Should say they 'are similar to each other'

**Response:**

Changes made to line 222- inclusion of article “a” in front of few

Line 354 (in edited manuscript) has been rephrased **from**: *The revised weights for leprosy disability grade 0 and grade 1 are not drastically different, which implies that the leprosy patients without any visible disability also experience comparable suffering*

**to** : *The revised weights for leprosy disability grade 0 are near similar to grade 1 weights , which implies that the leprosy patients without any visible disability also experience comparable suffering*
